# Large-Area and High-Throughput PDMS Microfluidic Chip Fabrication Assisted by Vacuum Airbag Laminator

**DOI:** 10.3390/mi8070218

**Published:** 2017-07-12

**Authors:** Shuting Xie, Jun Wu, Biao Tang, Guofu Zhou, Mingliang Jin, Lingling Shui

**Affiliations:** Institute of Electronic Paper Displays, South China Academy of Advanced Optoelectronics & Joint International Research Laboratory of Optical Information of the Chinese Ministry of Education, South China Normal University, Guangzhou 510006, China; stxie@m.scnu.edu.cn (S.X.); 2006wujun1999@163.com (J.W.); tangbiao@scnu.edu.cn (B.T.); guofu.zhou@m.scnu.edu.cn (G.Z.)

**Keywords:** large-area, microfluidic devices, fabrication, vacuum airbag laminator

## Abstract

One of the key fabrication steps of large-area microfluidic devices is the flexible-to-hard sheet alignment and pre-bonding. In this work, the vacuum airbag laminator (VAL) which is commonly used for liquid crystal display (LCD) production has been applied for large-area microfluidic device fabrication. A straightforward, efficient, and low-cost method has been achieved for 400 × 500 mm^2^ microfluidic device fabrication. VAL provides the advantages of precise alignment and lamination without bubbles. Thermal treatment has been applied to achieve strong PDMS–glass and PDMS–PDMS bonding with maximum breakup pressure of 739 kPa, which is comparable to interference-assisted thermal bonding method. The fabricated 152 × 152 mm^2^ microfluidic chip has been successfully applied for droplet generation and splitting.

## 1. Introduction

Microfluidics is also known by the names of microfluidic chip, lab on a chip (LOC) or micro-total-analysis-system (µ-TAS), which refers to building a micro-scale chemical or biological “lab” on a chip that is a few square centimeters in size [1,2]. A microfluidic chip contains a micro-channel network that allows the control of fluids to form a miniaturized chemical or biological “lab” where sample preparation, separation, detection, and other functions could be carried out [3,4]. Fabrication of stable and reliable microfluidic chips with various sizes is critical for their applications in different areas. Currently, many technologies aiming for functional chip fabrication in small size have been developed to fabricate microfluidic chips based on typical Micro-electromechanical Systems (MEMS) technologies [5,6,7,8,9]. When microfluidics is applied in the fields of emulsification [10], environmental analysis [11,12], and food safety [13,14], cheap and efficient technology for fabricating large area microfluidic chips becomes a bottleneck according to the limits of typical MEMS technologies. Therefore, it is necessary to find new technologies for large-area and high-throughput microfluidic chip fabrication, especially for industrialization.

Polydimethylsiloxane (PDMS) is the most popular material used to make microfluidic chips for both academic research (in biomedical, analytical, and biotechnological areas) and industrial production [8,15,16,17]. This is mainly attributed to the inherent properties of PDMS, such as optical transparency, biocompatibility, gas permeability, easy fabrication, low cost, and wide availability. On the other hand, soft-lithography technique has also enabled the widespread use of PDMS materials and opened up the era of PDMS-based microfluidics [18,19]. The fabrication of microfluidic devices typically includes structural design, mask fabrication, mold fabrication and replication, chip alignment, and bonding.

One of the key and difficult process challenges is how to align and bond the flexible PDMS sheet to another sheet of either hard glass or flexible PDMS to make a sealed chip. Precise alignment without bubble generation is mandatory for handling devices with flexible sheet. Up to now, there has been limited technical development focusing on such issues from the literature. Vacuum airbag laminator (VAL) is a commonly used piece of equipment in the liquid crystal display (LCD) production line. Two substrate sheets are respectively sucked onto top and bottom stages with vacuum of 0.01 bar. Typically, the alignment precision of 100 μm can be achieved for a 400 × 500 mm^2^ display panel [20,21]. According to the vacuum environment, bubble generation is also avoided. So, we chose the VAL equipment to pre-bond a large-area PDMS sheet to another glass or PDMS sheet without bubble generation.

In addition, various issues like bonding strength, interfacial stress, microchannel fidelity, solvent compatibility, surface chemistry, and optical properties of the bonded microfluidic chips should all be taken into account for the selection of appropriate bonding methods. So far, a variety of bonding technologies have been developed to seal PDMS-based microfluidic chips, such as thermal bonding [22], solvent bonding [23], plasma-aided bonding [24,25,26], adhesive bonding [27,28], and ultrasonic welding [29,30]. However, the limitations of either fabrication size or materials selectivity are obvious for these methodologies. Thermal bonding has been frequently chosen according to its simple process and high bonding strength, which is also suitable for larger-area device processing.

In this work, the combination of VAL and thermal bonding has been applied for large-area PDMS microfluidic chip fabrication. Optimal parameters of PDMS components (mass ratio of the pre-polymer to the curing agent), VAL alignment and pre-bonding, and thermal annealing process were investigated. The bond strength was evaluated by the peel, leak, and burst tests. The fabricated 152 × 152 mm^2^ microfluidic chips were successfully used to create and split microdroplets.

## 2. Materials and Methods

### 2.1. Materials

The glass substrate with thickness of 0.7 mm was purchased from Shenzhen Laibao Hi-tech Co. Ltd., Shenzhen, China. Deionized water (18.2 MΩ cm at 25 °C) was prepared by a water purification system (Water Purifier, Sichuan, China). The PDMS (Sylgard 184) package consisted of a base, and curing agent was purchased from Dow Corning Corporation (Midland, MI, USA). SU-8 3050 and its developer propylene glycol methyl ether acetate (PGMEA, 99%) were respectively purchased from MicroChem (Westborough, MA, USA) and Aladdin (Shanghai, China). N-hexadecane (99%) and sorbitane monooleate (Span 80, 99%) were purchased from Acros Organics (Geel, Belgium) and Aladdin (Shanghai, China), respectively. 1H,1H,2H,2H-perfluorodecyltrichlorosilane (FDTS, 96%) was purchased from Sigma Aldrich (Shanghai, China).

### 2.2. Fabrication of SU-8 Mold on Glass

**Figure 1:**
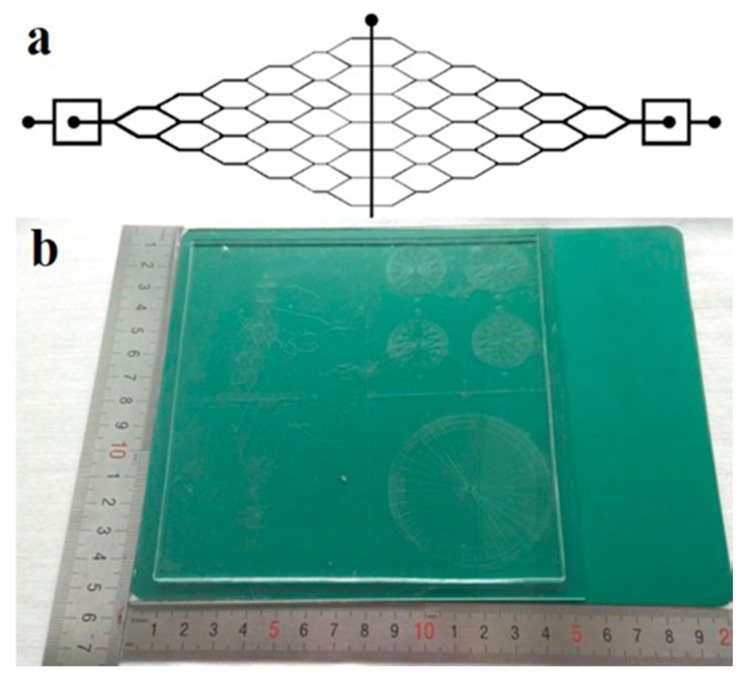
(**a**) Schematic drawing of the microfluidic device design; (**b**) Photograph of one fabricated large-area microfluidic plate with multiple chips.

Microfluidic channels on PDMS were fabricated using standard soft replication. According to the large-area device, the photomask and mold were also fabricated using glass substrate with the same size. Figure 1 shows the geometry of the microfluidic device. The microchannel shown in Figure 1a is a continuous Y-shaped microchannel network. It includes a vertically-tagged T-junction, and multiple tree-like Y-joints downstream microchannels. The width of the T-junction channel is 750.0 μm, and the width of the microchannel after each junction was sequentially decreased to half of the previous one. The smallest channel of the Y-shaped microchannel is about 11.7 μm. The microchannel length between two Y-joints is 2.7 mm. The whole length of the designed channel is 22.9 mm. The mold was fabricated on SU-8 3050 on a mother glass using standard lithography. Briefly, the photomask was obtained by transferring the CAD (computer-aided design) drawing onto a chrome plate. The mold glass substrate was chemically cleaned by immersing in piranha solution (H_2_SO_4_/H_2_O_2_ 3:1, *v*/*v*) for 10 min to remove organic impurities, and rinsed using deionized (DI )water and dried using nitrogen. Subsequently, the negative photoresist of SU-8-3050 was spin-coated on the glass, soft baked at 95 °C for 2 min, exposed for 150 s using an aligner (URE-2000/35, Chinese Academy of Sciences, Beijing, China), post-baked on a hotplate (EH20B, LabTech, Beijing, China) at 95 °C for 3 min, developed in PGMEA for 3 min, and then hard-baked at 150 °C for 30 min to obtain the photoresist patterned glass mold. Afterwards, FDTS coating was formed by vapor deposition [31].

### 2.3. Fabrication of PDMS Microfluidic Chips 

The PDMS substrate was prepared by initially mixing the pre-polymer solution with the curing agent at a certain mass ratio. The mixture was then degassed in a vacuum chamber for 30 min. Subsequently, the mixture was poured onto the prepared mold and cured in an oven (EH20B, LabTech, Beijing, China) at 90 °C for 30 min to replicate the microfluidic patterns from mold to PDMS. After peeling the PDMS sheet from the mold, holes were punched through for the in-chip and out-chip fluidic interconnection. The depth of the obtained microchannel is approximately 38 μm.

### 2.4. Bonding Procedure

**Figure 2:**
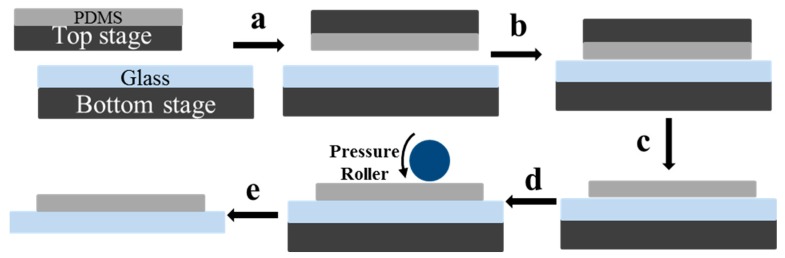
Schematic of the Vacuum airbag laminator (VAL) aligning and pre-bonding process: (**a**) flipping well-positioned top plate to the middle of the bottom plate; (**b**) aligning and laminating the flexible top sheet to the bottom plate; (**c**) releasing the polydimethylsiloxane (PDMS) sheet from the top stage; (**d**) pre-bonding the PDMS–glass chip by pressure roller; and (**e**) releasing the pre-bonded chip from the bottom stage.

The bonding process contains two major steps: VAL aligning and pre-bonding, and thermal annealing. Figure 2 shows the schematic illustration of the VAL aligning and pre-bonding steps. Before pre-bonding, the glass substrate was chemically cleaned by immersing in piranha solution (H_2_SO_4_/H_2_O_2_ 3:1, *v*/*v*) for 10 min, rinsed using DI water, and dried using N_2_, and the PDMS sheet was also thoroughly rinsed using DI water and dried using N_2_. Then, the PDMS sheet and glass substrate was positioned and attached on the top and bottom vacuum stages, respectively. The vacuum of top and bottom stages was respectively 0.01 and 0.008 bar, respectively. The top stage holding the PDMS sheet was then flipped (Figure 2a) and scrolled down to the appropriate position, and moved slowly to the right position for alignment from both *x*- and *y*-axis sides (Figure 2b). As soon as the alignment precision requirement was satisfied, two sheets were put together face-to-face (Figure 2c). The alignment precision was about 100 μm. Then, the PDMS sheet was released from the top plate by applying positive pressure to the top stage (Figure 2c). A pressure roller was then applied to the surface to press the PDMS and glass for pre-bonding (Figure 2d). The roller pressure was set at 0.15 MPa and the rolling velocity was 10 mm/s. The pre-bonded microfluidic chip was then released from the bottom stage by applying a positive pressure (Figure 2e). The prepared chip was then ready to be used directly or put into an oven for further thermal bonding.

### 2.5. Bond Strength Analysis

**Figure 3:**
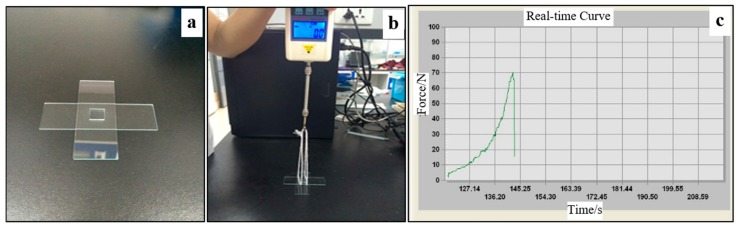
The bonding strength analysis: (**a**) the tested PDMS chip sample; (**b**) the home-made device with a digital tubular tensiometer; and (**c**) the software interface showing the pressure distribution applied on the contact surface between the two surfaces.

To characterize the bonding strength, peel and delamination tests were conducted. A home-made device was used to measure the force required to physically separate the patterned PDMS sheet and the glass substrate, as shown in Figure 3. Before testing, a PDMS substrate (10 × 10 × 1.5 mm^3^) was ordinarily bonded with a glass slide by VAL pre-bonding with or without thermal bonding. For comparison, the other surface of the PDMS was bonded with another glass substrate by the normal plasma-aided bonding [15]. Figure 3a exhibits a PDMS chip sample. The force was measured using the method as described in references [32,33], using the home-made device with a digital tubular tensiometer (ALIYIOI, Wenzhou Yiding Instrument Manufacturing Co. Ltd., Wenzhou, China) as shown in Figure 3b. Figure 3c shows a typical pressure versus time curve during the measurement. Because the force to separate the plasma-aided bonding was higher than that of the thermal bonding, the thermally-bonded glass slide would be first peeled off. Figure 3c provides details of the pressure distribution applied on the contact surface between the two surfaces, provided by the software. The maximum value was counted as the bond strength of the PDMS and glass. In order to secure the reproducibility, the tests were performed in triplicate.

### 2.6. Leak Test

Leak test was conducted to assess the sealing efficiency of the large-area PDMS–glass chips. 0.5 *w*/*w* % Rhodamine B solution was pumped into the microchannels at different flow rates using a syringe pump (KDS 200, Kd Scientific, Hongkong, China). The flow rate at which the dyed liquid started to squeeze out of the microchannel is regarded as the beginning of the leakage.

### 2.7. Optical Measurement of Microfluidic Chip

To prove the validity of this method, the fabricated 152 × 152 mm^2^ microfluidic chip was tested. Two immiscible liquid phases were introduced into the microchannels via the inlets through polytetrafluoroethylene (PTFE) tubes by two syringe pumps (KDS 200, Kd Scientific, Hongkong, China). The fluidic flow behavior was visualized and recorded using an inverted optical microscope (Olympus IX2, Tokyo, Japan) equipped with a high-speed camera (Phantom Miro M110, Vision Research Inc., Wayne County, NC, USA).

## 3. Results and Discussion

### 3.1. Thermal Bonding of PDMS–Glass

Bonding quality is a key factor in achieving reliable microfluidic chips. Therefore, optimal parameters of thermal bonding were investigated. Typically, thermal bonding is performed at a temperature higher than the glass transition temperature (*T*_g_) of investigated materials. *T*_g_ of PDMS is ~120 °C [31], and the glass substrate can survive up to 600 °C without obvious property change. Therefore, the bonding temperature of 120–260 °C has been tested for optimizing the bonding process.

**Figure 4:**
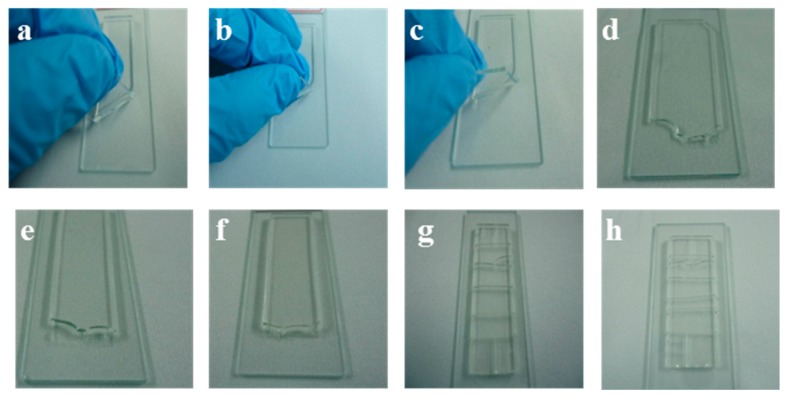
The peeling-off experiments at different temperatures: (**a**) 120 °C; (**b**) 140 °C; (**c**) 160 °C; (**d**) 180 °C; (**e**) 200 °C; (**f**) 220 °C; (**g**) 240 °C; and (**h**) 260 °C.

A PDMS substrate (10 × 10 × 1.5 mm^3^) was manually put onto a glass slide surface and heated in an oven at different temperatures (*T*) for 4 h to achieve thermal bonding. At the same time, plasma-aided bonding was also carried out for comparison. Peel test was carried out to analyze the bonding strength and efficiency at different temperature. From the photos shown in Figure 4a–h, we can find that the quantity of residual PDMS on the glass surface bonded at 220 °C is the most after peeling off process, which is comparable to the plasma-aided bonding.

**Figure 5:**
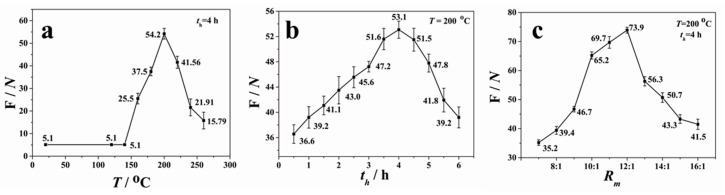
The variation of bonding force with (**a**) temperature (*T*); (**b**) holding time (*t*_h_) at 200 °C; and (**c**) the mass ratio of pre-polymer to curing agent (*R*_m_) of PDMS substrate.

In addition, the force required to separate the bonded PDMS and glass sheets after thermal bonding was measured to evaluate the bond strength. The force (*F*) was obtained by averaging three measurements under the same conditions. Figure 5a presents the measured force as a function of the annealing temperature (*T*) (the mass ratio of the pre-polymer to the curing agent was 10:1). When the temperature was below 150 °C, *F* was maintained at about 51 kPa (5.1 N divided by 10 × 10 mm^2^). With the increase of *T*, *F* increased when *T* was in the range of 150–200 °C; it decreased when *T* was higher than 200 °C. Thus, the maximum force was achieved at *T* = 200 °C, with the value of about 542 kPa.

The effect of holding time on the bonding efficiency was studied at *T* = 200 °C. The pre-bonded devices were put in the oven heated up to 200 °C and held for different periods of time (*t*_h_). As shown in Figure 5b, the bonding strength increased with holding time when *t*_h_ was in the range of 0.5–4 h. When *t*_h_ > 4 h, the adhesion force gradually reduced. The adhesion force was about 531 kPa when *t*_h_ = 4 h, which is consistent with the result of Figure 5a. The cracking and decreased adhesion force at higher temperature may be attributed to the oxidation or decomposition of PDMS.

Figure 5c shows the profile of the measured force between the PDMS–glass substrates with different PDMS compositions by varying the mass ratio (*R*_m_) of the pre-polymer to the curing agent. The variation of mass ratios means different degrees of cross-linking of PDMS. The lower the mass ratio is, the lesser the degree to which the PDMS molecules are cross-linked, and the softer the PDMS sheet is. Varying the crosslink density in the polymer network allows us to tune the mechanical and optical properties of the obtained PDMS substrate to adapt to different applications. As seen in Figure 5c, the bonding strength increased with *R*_m_ when *R*_m_ < 12:1, then sharply reduced when *R*_m_ was changed from 12:1 to 13:1. After that, the force slowly decreased with *R*_m_. Therefore, the maximum force of 739 kPa was obtained under the conditions of *R*_m_ = 12:1, *T* = 200 °C, and *t*_h_ = 4 h, which is higher than the plasma-aided bonding and interference-assisted thermal bonding [29,32]. Such parameters were then used commonly for the rest of experiments in this work.

### 3.2. Large-Area Microfluidic Device Fabrication Using VAL

The vacuum airbag laminator plays an important role in the LCD panel production line. Display devices with areas up to 400 × 500 mm^2^ (G2.5 of LCD line) have been fabricated in our lab using VAL, with the alignment precision of about 100 µm. Both LCDs and microfluidic devices are composed of glass and flexible substrates. Therefore, the use of VAL has predetermined advantages for microfluidic device fabrication. The possible fabrication size can hereby be comparable to the maximum LCDs; for instance the G11 line (3000 × 3320 mm^2^), which is highly valuable for industrialization.

VAL is a technology manipulated at room temperature, which can not only improve the alignment speed and accuracy but also avoid bubble generation, as described in the Materials and Methods session. To assess the sealing efficiency of the large-area PDMS–glass chips, leak tests were conducted by pumping 0.5 *w*/*w* % Rhodamine B solution into the whole microchannels from the inlet at different flow rates. The flow rate was increased from 100 μL∙h^−1^ until the beginning of the leakage. When the dyed liquid started to squeeze out of the microchannel, we counted the flow rate as the beginning of the leakage. The pressure (∆*P*, Pa) between inlet and leakage position of the channel was calculated by Poiseuille’s law: ∆*P* = *QR*_hy_, where *Q* (m^3^∙s^−1^) is the applied volume flow rate and *R*_hy_ (kg∙s^−1^·L^−^^4^) is the hydrodynamic flow resistance. Assuming rectangular cross-section of the microchannels, *R*_hy_ could be approximately expressed as: *R*_hy_ ≈ *ηLC*^2^*A*^−3^, where *η* (Pa∙s) is the viscosity of the Rhodamine B solution, *L* (m) is the microchannel length, *C* (m) and *A* (m^2^) are the perimeter and area of the cross-section. Based on the tree-like microchannel arrangement, the pressure at each Y-junction block was calculated and then added to obtain the total pressure drop over the microfluidic network, which was about 30.5 kPa.

**Figure 6:**
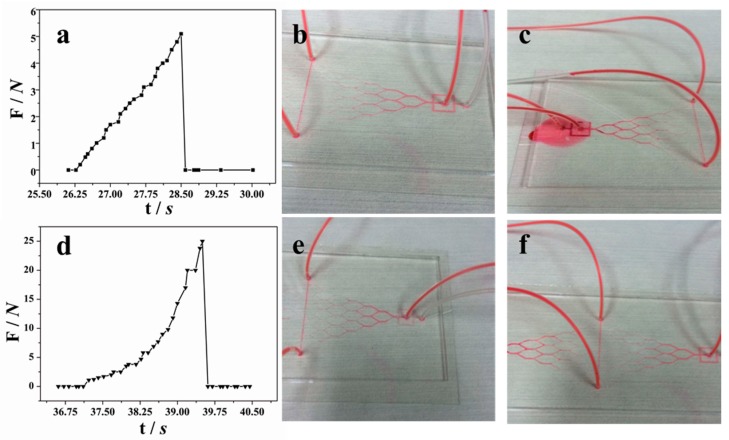
Comparison of adhesion force (**a**,**d**) and leakage flow rates (**b**,**c**,**e**,**f**) when PDMS–glass devices were bonded by VAL pre-bonding without thermal bonding (**a**–**c**), and the combination of VAL pre-bonding and thermal bonding at 200 °C for 4 h (**d**–**f**). The applied flow rates were: (**b**) 200 μL·h^−1^; (**c**) 250 μL·h^−1^; (**e**) 200 μL·h^−1^; and (**f**) 1000 μL·h^−1^, respectively.

Figure 6 shows the peel and leak tests of the fabricated PDMS–glass using only VAL pre-bonding (Figure 6a–c) and the combination of VAL pre-ponding and thermal bonding (Figure 6d,e). The only VAL pre-bonding could withstand a flow rate of 200 μL·h^−1^ (Figure 6b) with the maximum bonding strength of about 51 kPa (Figure 6a) which may not be enough for general microfluidic experiments. As seen in Figure 6c, the leakage started at the first joint where the sudden increase of flow velocity happened due to the large size change from 3 × 1.5 mm to 1 × 750 μm.

After thermal bonding treatment at 200 °C for 4 h, the average bonding strength reached 251 kPa, which is five times higher than those without thermal annealing, as seen from Figure 6d. The leakage flow rate was about 1000 μL·h^−1^, as shown in Figure 6e,f. Obvious bonding strength improvement was obtained by the thermal annealing.

After bonding, either one large-area multifunctional microfluidic chip or multiple small microfluidic chips cut from one large plate could be obtained at once. Therefore, the production speed is enhanced, and the price per chip is reduced.

### 3.3. Droplet Creation and Splitting in the Microfluidic Chip

**Figure 7:**
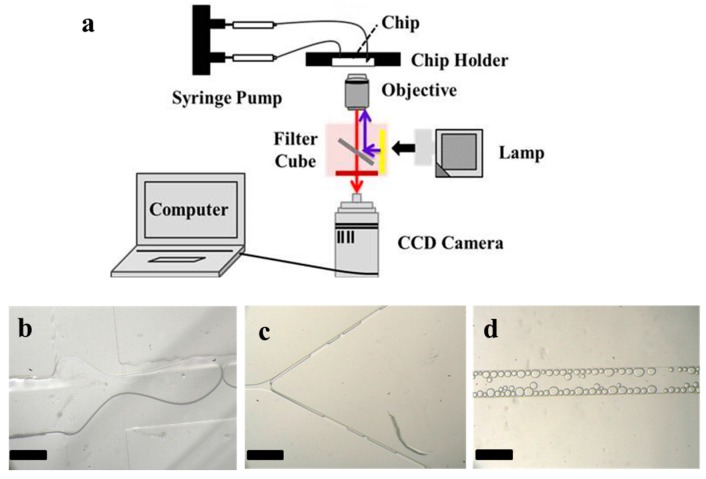
(**a**) Schematic illustration of the experimental set-up; (**b**) A snapshot of the droplet generation at the T-junction; (**c**) A snapshot of the droplet breakup at the Y-joint; (**d**) A snapshot of the droplets flow in the outlet channel after being broken at various ratio from the Y-joint. All scale bars in the figures are 500 μm.

To prove the validity of this method, the fabricated 152 × 152 mm^2^ microfluidic chip was tested. Two immiscible liquid phases of DI water and hexadecane with 3.0 *w*/*w* % Span 80 were introduced into the microchannel via the inlets through PTFE tubes by two syringe pumps. The schematic illustration of the experimental set-up is shown in Figure 7a. The geometry of the microfluidic device is shown in Figure 1, which includes a vertically-tagged T-junction and multiple tree-like Y-joints downstream microchannel networks. When the two immiscible phases met at the T-junction, the water phase broke into droplets dispersed in the organic continuous phase (Figure 7b). When the generated larger droplets flew through the downstream, they were cut into smaller ones according to the flow resistance distribution caused by the obstacle of the Y-joint channel, as shown in Figure 7c and Mov.S1. The average diameter and standard deviation of microdroplets in the outlet channel were, respectively, 90.36 μm and 8.4% when the oil and water flow rates was 600 μL·h^−1^ and 200 μL·h^−1^, respectively (Figure 7d). The fabricated 152 × 152 mm^2^ PDMS–glass microfluidic chip has been successfully applied for droplets creation and splitting.

## 4. Conclusions

In conclusion, a straightforward and efficient method for large-area PDMS–glass microfluidic chip fabrication has been proposed and verified. The vacuum airbag laminator plays a key role for flexible PDMS sheet aligning and pre-bonding with glass substrate without air bubble generation. The size of the bonded device is determined by the available size of the laminating machine. Thermal bonding has been applied to improve the bonding strength from 52 kPa to 739 kPa under the optimal conditions *R*_m_ = 12:1, *T* = 200 °C, and *t*_h_ = 4 h, which is comparable to those obtained from plasma-aided bonding and interference-assisted thermal bonding. The fabricated 152 × 152 mm^2^ PDMS–glass microfluidic chip has been successfully applied for droplet creation and splitting in the same device. Either one large-area multifunctional microfluidic chip or multiple small microfluidic chips cut from one large plate could be obtained at once by this fabrication method. The VALs can realize large-area hard-to-hard, soft-to-hard, and soft-to-soft device fabrication. Such a method has borrowed and combined existed technologies from matured LCD production line to the microfluidic area, making it easier for industrialization and commercialization.

## Supplementary Materials

The following are available online at www.mdpi.com/2072-666X/8/7/218/s1, Mov.S1: The process of generated bigger droplets cut into smaller ones when they flow through the downstream Y-junctions.
